# The Prevalence of Sarcopenia among Hunan Province Community-Dwelling Adults Aged 60 Years and Older and Its Relationship with Lifestyle: Diagnostic Criteria from the Asian Working Group for Sarcopenia 2019 Update

**DOI:** 10.3390/medicina58111562

**Published:** 2022-10-30

**Authors:** Jing Zhong, Wenqing Xie, Xiaoqin Wang, Xin Dong, Yihan Mo, Dan Liu, Xuemei Yao, Beibei Liu, Wenyu Deng, Yidong Su, Yusheng Li, Xiuhua Wang

**Affiliations:** 1 Deparment of Nephrology, The Third Xiangya Hospital, Central South University, Changsha 410013, China; 2 Deparment of Orthopedics, Xiangya Hospital, Central South University, Changsha 410008, China; 3 Department of Geriatrics, The Second Xiangya Hospital, Central South University, Changsha 410011, China; 4 Xiang Ya Nursing School, Central South University, Changsha 410013, China; 5 National Clinical Research Center for Geriatric Disorders, Xiangya Hospital, Central South University, Changsha 410008, China

**Keywords:** ageing, sarcopenia, community-dwelling older adults, prevalence, lifestyle

## Abstract

*Background and Objectives*: This study aims to detect the prevalence of sarcopenia in community-dwelling older adults in Hunan Province, discuss factors related to lifestyle, and provide a reliable basis for the prevention and treatment of sarcopenia. *Materials and Methods*: In this study, a total of 1040 community-dwelling adults ≥ 60 years were examined for sarcopenia using a cluster stratified random sampling method, which was defined using the diagnostic criteria recommended by the Asian Working Group for Sarcopenia (AWGS) from September 2019 to March 2020. Multivariate logistic regression analysis was applied to determine the correlation between sarcopenia and smoking, drinking, nutritional status, physical activity, and sleep quality. *Results*: A total of 27.1% of the older adults were diagnosed with sarcopenia, with rates of 26.2% in men and 25.2% in women. Multiple logistic regression showed that advanced age (OR = 2.480, 95% CI: 1.730, 3.553), the risk of malnutrition (OR = 2.085, 95% CI: 1.440, 3.019), and malnutrition (OR = 1.212, 95% CI: 0.304, 4.834) were risk factors for sarcopenia. No falls in the previous year (OR = 0.616, 95% CI: 1.885, 1.209), normal weight (OR = 0.228, 95% CI: 0.109, 0.475), overweight (OR = 0.030, 95% CI: 0.013, 0.069), moderate physical activity (OR = 0.593, 95% CI: 0.377, 0.933), or high physical activity (OR = 0.417, 95% CI: 0.230, 0.755) were identified as protective factors for sarcopenia. *Conclusions*: The prevalence of sarcopenia was high among older adults in the community in Hunan Province. In addition, we found that lifestyle is an important factor in sarcopenia.

## 1. Introduction

With the rapid development of medical technology and the continuous improvement of people’s living standards, the average life expectancy of people is generally extended, and the global older adult population is growing rapidly [1]. With increasing age, the function of various organs and systems of older adults declines, and these individuals suffer from a variety of chronic diseases. All these changes have extremely adverse effects on the health and prognosis of the diseases and represent a great challenge to public health, medical care, and home care [2]. During this process, each component of the body will change, and the most representative change is observed in skeletal muscle [3]. Studies have shown that after the age of 50 years, muscle mass continues to decrease at a rate of 10% every 10 years, and muscle strength decreases by 15% every 10 years after 70 [4].

The concept of “sarcopenia” was first proposed by the American scholar Osenberg in 1989 [5]. Given the increase in the ageing of the world’s population, sarcopenia is particularly prominent in older adults. Sarcopenia has gradually attracted the attention of the medical profession, especially geriatric researchers. In 2010, the European Working Group on Sarcopenia in Older People (EWGSOP) defined sarcopenia as a geriatric syndrome that can lead to the decline of functional status and quality of life in older adults that is characterised by progressive, generalised muscle loss and reduced muscle strength or physiological function related to ageing [6].

At present, due to the sharp increase in the older adult population, sarcopenia has become one of the increasingly serious public problems that threaten the health of older adults. The prevalence of sarcopenia in the older adult community ranges between 1% and 29%, and the prevalence of sarcopenia in older adults in nursing homes is 14%~33% [7]. The prevalence of sarcopenia in elderly individuals over 80 years is as high as 11%~55% [8]. Sarcopenia leads to decreased muscle function and restricted mobility in older adults. Furthermore, negative psychology of anxiety, depression, and reduced quality of life also occur in older adults with sarcopenia [9,10,11]. Hence, timely and accurate screening of sarcopenia in older adults is particularly important for its prevention and treatment.

The occurrence and development of sarcopenia are the results of multiple factors. Studies performed on the factors influencing sarcopenia revealed that demographic factors (age, sex, etc.), lifestyle (nutrition and exercise), disease factors (osteoporosis, weakness, diabetes, etc.), and psychological factors (depression and anxiety) are related to the occurrence and development of sarcopenia [12]. However, among the factors that contribute to the development of sarcopenia, lifestyle is an easy-to-change and controllable factor compared with the irreversible factors caused by ageing. Previous studies have also confirmed this notion. Chun-Wei Li et al. found that lifestyle interventions, such as nutrition and exercise, can significantly improve the muscle mass of patients with sarcopenia, reduce inflammation, restore their metabolic hormone levels, and reduce the risk of sarcopenia [13]. A meta-analysis showed that a U-shaped relationship exists between sleep time and the risk of sarcopenia. Both too much and too little sleep increase the risk of sarcopenia [14]. Our study explores the controllable risk factors for sarcopenia from the perspective of lifestyle factors, aiming to provide a basis for the prevention and control of sarcopenia in the elderly community to promote healthy ageing.

## 2. Materials and Methods

### 2.1. Study Population and Design

This study was conducted from September 2019 to March 2020. We selected older adults (age ≥ 60 years, living in the community for one year or more) as the research participants. The study sample was obtained from Hunan Province, which was divided into five regions: Eastern Hunan (Changsha, Zhuzhou, Xiangtan), Western Hunan (Xiangxi Tujia and Miao Autonomous Region, Huaihua, Zhangjiajie), Southern Hunan (Hengyang, Chenzhou, Yongzhou), Northern Hunan (Changde, Yueyang), and Central Hunan (Loudi, Shaoyang, Yiyang) by a cluster stratified random sampling method. Then, from the five regions, a prefecture-level city was identified where a random extract of 2~4 communities was selected to extract the required sample size. Inclusion criteria were as follows: (1) capable of completing the questionnaire survey, and (2) capable of completing the grip strength test and 6 m gait speed test. The exclusion criteria were as follows: (1) cannot stand alone or have pacemakers, heart stents, etc., (2) severe cognitive impairment, patients with mental diseases, terminal diseases, or severe acute and chronic diseases, and (3) severe visual or hearing impairment and inability to communicate or cooperate. A total of 1100 older adults were invited to participate in this study, of which 1040 patients agreed to participate in this study. Face-to-face interviews were conducted by professionally trained personnel. This research was approved by the Research Ethics Committee of Central South University (No: 2019020), and all research ethics were adhered to throughout the entire study.

### 2.2. Anthropometric Measures

Bioelectrical impedance analysis (BIA) has been used to estimate the total muscle or accessory bone muscle mass ASM. The BIA device does not directly measure the muscle mass but estimates the muscle mass based on the whole body′s electrical conductivity. At the same time, BIA can measure body fat, bone mass, visceral fat, and other indicators. AWGS 2019 recommends using DXA or multifrequency BIA (all height adjusted) to measure muscle mass in the diagnosis of sarcopenia [12]. The AWGS 2019 cutoffs for low muscle mass in sarcopenia diagnosis are as follows: <7.0 kg/m^2^ in men and <5.4 kg/m^2^ in women by DXA, and <7.0 kg/m^2^ in men and <5.7 kg/m^2^ in women by BIA. In addition, the European Working Group on Sarcopenia in Old People (EWGSOP) and EWGSOP2 also point out that BIA can be used as the main tool for a community screening of muscle mass and think that BIA-based muscle mass measurement may be preferable to DXA in terms of convenience and portability. The cutoff points of muscle mass measured by DXA and BIA were proposed, respectively [e.g., EWGSOP: DXA (male < 7.26 kg/m^2^, female < 5.50 kg/m^2^); BIA (male < 8.87 kg/m^2^, female < 6.42 kg/m^2^] [6,15]. An InBody S10 body composition analyser was used to determine the body composition (Biospace Co., Ltd, Gangnam-gu, Seoul, Korea). The following measurement criteria were employed: (1) fasting measurement, avoiding strenuous exercise 15 minutes before the test, (2) removing metal ornaments and mobile phones, exposing hands and feet, lying on the bed, (3) connecting 8 motor points to hands and feet, and (4) ability to maintain the same posture and not talk until the end of the measurement.

### 2.3. Measurement of Handgrip Strength (HG)

The dominant hand grip strength is measured with an electronic grip strength meter (Jamar, Los Angeles, CA, USA). Before the test, adjust the grip distance of the grip according to the size of the patient’s palm. During the test, the patient took the sitting position, bent the elbow 90°, repeated 3 times with an interval of 1 min, and recorded the maximum value (kg) for further analysis [16].

### 2.4. The Chair Standing Test (CRT)

The chair standing test was used to measure the strength of the leg muscles of older individuals. The method measured the time older adults sitting on a chair spent from sitting to standing 5 times. In this process, older adults should try not to use their arms for assistance [15].

### 2.5. Measurement of 6 m Gait Speed

The study participants were asked to walk starting from a standing position and straight for 10 meters at their normal gait speed. The time taken to traverse the last 6 m was obtained. The test was performed twice, and the average was taken [12].

### 2.6. Diagnostic Criteria for Sarcopenia

This study employed the AWGS 2019 updated diagnostic criteria as follows: male muscle mass < 7.00 kg/m^2^, female < 5.70 kg/m^2^, male grip strength < 28 kg, female grip strength < 18 kg, and 6 m gait speed < 1 m/s. If a decrease in muscle mass and muscle strength or gait speed was noted, sarcopenia was diagnosed [12].

### 2.7. Assessment of Covariates General Information

The following covariates were obtained through face-to-face interviews: gender, age, marital status, occupation, place of residence, nationality, education level, medical insurance, income level, smoking, drinking, falling, and diseases, such as hypertension, diabetes, coronary heart disease, stroke, hyperlipidemia, and other complications. The height and weight of all participants were measured, and the Body mass index (BMI) was calculated and defined as the weight (kg) divided by height (m)^2^. BMI < 18.5 kg/m^2^ indicated underweight, a BMI of 18.5–24.9 kg/m^2^ indicated normal weight, and BMI > 25 kg/m^2^ indicated overweight.

### 2.8. Lifestyle Information

Factors related to lifestyle, such as the history of smoking, drinking, nutritional status, physical activity, and sleep quality, were also obtained.

Short-form Mini Nutritional Assessment (MNA-SF) consists of six highly relevant items extracted from the MNA scale by Rubenstein et al. [17] in 2001. The specific assessment contents are as follows: (1) BMI < 23, (2) Recent weight loss > l Kg, (3) acute disease or stress, (4) bedridden or not, (5) dementia or depression, and (6) loss of appetite or difficulty in eating. The total score for the entire scale is 14 points, while 12–14 points indicate normal nutritional status, 8–11 points indicate risk of malnutrition, and <7 points indicate malnutrition. Cronbach’s α is 0.843 [18].

The Elderly Physical Activity Scale (PASE) was used to evaluate the physical activity of an elderly individual in the past week. The total score ranged from 0~360, and the weights of different activities were different. The scores of physical activity of the older adults were calculated according to the weights of various activities, and they were divided into high, medium, and low levels. A physical activity score less than 50% of the overall average indicated a low physical activity level. A physical activity score greater than or equal to 50% but less than 150% of the average of overall physical activity was identified as middle-level physical activity. A physical activity score greater than 150% of the average overall physical activity was identified as a high level of physical activity [19].

Using the Self-Rating Scale of Sleep (SRSS) to assess sleep problems in older adults, a total score of 22 points indicated normal sleep, 23~29 points indicated mild sleep problems, 30~39 indicated moderate sleep problems, and 40~50 indicated severe sleep problems [20].

### 2.9. Statistical Analysis

SPSS 18.0 statistical software was used to analyse the data in this study. If the measurement data conformed to a normal distribution, the mean ± standard deviation was reported. If the test of normality was not met, the median and interquartile range were reported. The frequency (composition ratio) was used to report count data. Two independent sample t-tests (or Mann–Whitney U tests) were used for comparison between the two groups. Analysis of variance (or Kruskal–Wallis H test) was used for comparison between the three groups. Chi-square tests were used for the comparison of categorical variables. Multivariate logistic regression analysis was used to explore the relationship between sarcopenia and influencing factors. Odds ratios (ORs) and 95% confidence intervals (CIs) were used to express the relationship between sarcopenia and its influencing factors. *p* < 0.05 was considered statistically significant.

## 3. Results

### 3.1. Description of the Study Population

This study involved 1040 older adults. In total, 66.6% of participants were female, with an average age of 70.39 ± 7.40 years.

### 3.2. Prevalence of Sarcopenia

Table 1 shows the comparative analysis of the prevalence and related indicators of sarcopenia in older adults based on the diagnostic criteria of the Asian Sarcopenia Working Group. In this study, the overall prevalence of sarcopenia was 27.1%. The prevalence in women was 26.6%, and the prevalence in men was 25.2%. No statistically significant differences were noted between men and women (*p* > 0.05). Compared with the non-sarcopenia group, participants with older age, low education level, no spouse, history of falls, smoking history, alcohol consumption, and low BMI were more likely to suffer from sarcopenia (*p* < 0.05). In addition, compared with the non-sarcopenia group, low levels of physical activity, risk of malnutrition or malnutrition, and sleep disorders were more common in the sarcopenia group, and the difference was statistically significant (*p* < 0.05).

### 3.3. Effect of Age on the Prevalence of Sarcopenia in Older Adults and Related Indicators

According to Table 2, among different age groups, the skeletal muscle mass index (SMI), grip strength, BMI, and body fat percentage of the older adults in the sarcopenia group were lower than those in the non-sarcopenia group, whereas the 6-metre pace and CRT were higher than those in the non-sarcopenia group. Additionally, the differences in the prevalence of sarcopenia, SMI, grip strength, 6-metre pace, CRT, BMI, and body fat percentage were statistically significant (*p* < 0.05).

### 3.4. Effect of Gender on the Prevalence of Sarcopenia in Older Adults and Related Indicators

It can be seen from the descriptive data in Table 3 that among males and females, the SMI, grip strength, BMI, and per cent body fat of the elderly in the sarcopenia group are lower than those in the non-sarcopenia group, while the 6-meter pace and CRT are higher than those in the non-sarcopenia group. The incidence of sarcopenia in females was higher than in males, but the difference was not statistically significant (*p* > 0.05). In the population with sarcopenia, the SMI and grip strength of females were significantly lower than those of males, and the 6-meter walking speed and CRT were significantly higher than those of males. There were significant differences between males and females in SMI, grip strength, 6-meter pace, CRT, BMI, and per cent body fat (*p* < 0.05).

### 3.5. Factors Associated with Sarcopenia

The results of the logistic regression analysis are shown in Table 4. The study found that advanced age (OR = 2.480, 95% CI: 1.730, 3.553), the risk of malnutrition (OR = 2.085, 95% CI: 1.440, 3.019), and malnutrition (OR = 1.212, 95% CI: 0.304, 4.834) were risk factors for sarcopenia. No falls within the previous year (OR = 0.616, 95% CI: 1.885, 1.209), normal weight (OR = 0.228, 95% CI: 0.109, 0.475), overweight (OR = 0.030, 95% CI: 0.013, 0.069), moderate physical activity (OR = 0.593, 95% CI: 0.377, 0.933), and high physical activity (OR = 0.417, 95% CI: 0.230, 0.755) were identified as protective factors for sarcopenia.

## 4. Discussion

Sarcopenia is the result of ageing and disease. Its influencing factors include age growth, reduced exercise, poor nutrition, hormone level disorder, neuromuscular regulation imbalance, a long-term increase of inflammatory cytokines in the elderly, etc. In addition, different populations, races, genetic backgrounds, and living environments will also have different effects on sarcopenia. According to the diagnostic criteria of AWGS updated in 2019, we found that sarcopenia is common in older adults in the communities of Hunan Province. In this study, the prevalence of sarcopenia was 27.1%. Specifically, the prevalence in women was 26.6%, and the prevalence in men was 25.2%. The difference between the sexes was not significant. In comparison, cross-sectional studies in Korea also showed that the prevalence of sarcopenia in older adults was 20.1% in men and 29.2% in women using the same diagnostic criteria, and the difference between the sexes was not significant [21]. The prevalence of sarcopenia in this study was greater than that reported in other similar studies [22,23], which may be attributed to the notion that the 2019 AWGS diagnostic criteria can more comprehensively screen the sarcopenia population [12].

Moreover, because of the current diagnostic criteria, as well as the race, social and cultural backgrounds, and lifestyle differences of the study population, the rate of sarcopenia is also inconsistent. A survey of 408 older adults in Taiwan showed that the prevalence of sarcopenia was 7.8% based on the diagnostic criteria of the EWGSOP, while the prevalence of sarcopenia was 4.1% when using the International Working Group on Sarcopenia (IWGS) criteria [24]. A survey of 407 older Chinese adults with an average age of 81 years revealed a sarcopenia prevalence of 44% in men and 26% in women, and the prevalence in men was greater than that in women [25]. A systematic review survey showed that the prevalence rate of sarcopenia was 1%~29% in the community, 14%~33% in people living in nursing homes for a long time, and 10% in older adults in the emergency department [26]. The incidence of sarcopenia exhibits a relatively high level. Although the prevalence of sarcopenia in the same cohort is different, as the ageing population increases, the incidence of sarcopenia will gradually increase.

The prevalence of sarcopenia in women aged 60–69, 70–79, and over 80 years was 19.5%, 28.9%, and 51.1%, respectively, and age was an independent factor influencing sarcopenia in our study. This finding may be explained by the fact that after the age of 50, skeletal muscle mass decreases at a rate of 1% to 2% per year. Between the ages of 50 and 60, muscle strength decreases at a rate of 1.5% per year and begins to decline at a rate of 3% after the age of 60 [24]. Therefore, with increasing age, the prevalence of sarcopenia also increases. Han et al. found that the prevalence of sarcopenia in women aged 60–69, 70–79, and over 80 years old was 3.9%, 11.9%, and 16.7%, respectively, and age was an important risk factor for sarcopenia [25]. The study by Cheng et al. revealed the differences in BMI, SMI, and fat among older adults of different ages. Specifically, as the age increases, the lower the SMI, the higher the BMI and body fat percentage [27]. The two studies are consistent with the results of our study. On the other hand, increasing age will lead to changes in hormone levels, such as growth hormone, oestrogen, androgens, and insulin, and changes in these hormones are involved in the pathogenesis of sarcopenia [28]. It is unquestionable that age is one of the decisive factors involved in the development of sarcopenia.

Although it is not found in this study that there is a correlation between the prevalence of sarcopenia and gender, the research results show that the prevalence of female sarcopenia is higher than that of males. The prevalence of female sarcopenia is 26.6%, and the prevalence of male sarcopenia is 25.2%. In addition, The SMI, grip strength, and BMI of men were higher than those of women, while the CRT and body fat percentage were significantly lower than those of females. It can be seen that female muscle health is significantly weaker than males. The reason for this result may be that skeletal muscle cells express estrogen located in the cell membrane, cytoplasm, and nuclear membrane β receptors, and inhibiting estrogen can directly affect muscle quality. With the increase in age, the estrogen level of elderly females decreases significantly, which can explain why the incidence of sarcopenia in elderly females is significantly higher than in males. In addition, females live longer, so females have a higher risk of sarcopenia, which will bring bigger public health problems. Therefore, we should pay more attention to the prevalence of female sarcopenia.

This study found that in the past year, the history of falls in the sarcopenia group was greater than that in the non-sarcopenia group and that no history of falls in the past year was a protective factor for sarcopenia. This finding is similar to that of Woo [29] and Gadelha et al. [30]. Previous studies have shown that because muscle mass and muscle strength are important components in regulating the relationship between falls and ageing, sarcopenia can lead to a decrease in muscle mass and muscle strength in older adults; thus, their risk of falls increases [31]. Furthermore, older adults have a fear of falling, especially those who have a history of falls, given that falls can lead to disability and reduced physical activity, resulting in decreased muscle function [31,32]. In addition, some older adults who have fallen have already experienced limb dysfunction, and their physical activity is restricted, resulting in restricted muscle function [31]. Disuse of the limbs can cause fat infiltration, which affects the transformation of muscle fibres from type II to type I, resulting in impaired contractility, loss of muscle mass, and decreased muscle strength [33,34]. In summary, in the management of sarcopenia, we should focus on older adults who have a history of falls.

The current study found that aerobic exercise and resistance exercise can effectively improve the muscle mass and muscle strength of older adults [35,36]. However, the elderly cannot perform high-intensity physical training, and their daily physical activities should be assessed. Some studies have shown that low-level physical activity is a risk factor for sarcopenia [37], which can induce anabolic resistance, protein synthesis after meals, insulin sensitivity, and leg muscle mass in older adults [38]. The results of this study also revealed that more older adults exhibited high levels of physical activity in the non-sarcopenia group compared with the sarcopenia group, and the level of physical activity was an independent factor for sarcopenia. This result is similar to that of MK Karlsson et al. [39]. The above studies have shown that physical exercise has a positive effect on muscles, but Shan Hai’s research shows that sarcopenia is not related to physical activity [40]. The reason for this finding is that maintaining the muscle strength of older adults may depend on specific types of physical exercise [41]. Therefore, the relationship between sarcopenia and the intensity of physical activity is worthy of further study. Even if the relationship between the two factors is disputed, the study also found that lifestyle and physical health in the early stages of life determine the speed at which the muscle strength of older adults decreases [42]. Therefore, we still need to carry out appropriate physical exercises for middle-aged and older adults to prevent further decline in muscle function.

This study shows that good nutritional status is a protective factor for sarcopenia. This finding is similar to that noted in previous research by Xu et al. [43]. Many lines of evidence also show that inadequate protein intake, energy, certain micronutrients, and malabsorption are risk factors for sarcopenia [44,45]. Food intake of older adults decreases with age, and studies have shown that to maximise the stimulation of muscle protein synthesis in older adults, a greater relative protein intake is required. The European Society of Clinical Nutrition and Metabolism recommended that the protein intake of older adults under normal conditions should be greater than 1.2–1.5 kg/d, that of older adults with malnutrition or chronic diseases should be greater than 1.5 kg/d, and that of young people should only be greater than 0.8 g/d [46,47,48]. Additionally, the speed of muscle protein synthesis in older adults is reduced, leading to muscle loss. In addition, because older adults often suffer from a variety of chronic diseases, they are prone to injury and take a long time to recover from injury, which leads to protein synthesis and decomposition changes and muscle atrophy [49]. In summary, ensuring that older adults in the community are in a good nutritional state is of great significance to the prevention and treatment of sarcopenia.

This study found that sleep status is related to sarcopenia. This finding is similar to previous research on older adults in Chinese communities [50]. The mechanism between sleep and sarcopenia is currently unclear. The possible mechanisms are as follows: The decline in sleep time and sleep quality caused by ageing will accelerate proteolysis, change body composition, and increase the risk of insulin resistance. These factors may be involved in the pathogenesis of sarcopenia. In addition, sleep disorders will reduce muscle protein synthesis and enhance muscle protein degradation [14]. A common mechanism may exist between sarcopenia and sleep, and it is necessary to further explore the potential role of sleep in the occurrence of sarcopenia.

The limitations of this study included the following: First, this study excluded older adults who were bedridden or had impaired cognitive function. There may be deviations in the estimation of the prevalence of sarcopenia. Future studies should include all older adults as much as possible to increase the representativeness of the sample. Second, covariates, such as physical activity, nutritional status, and sleep status, were obtained through self-report questionnaires, potentially leading to recall bias. Finally, because of the nature of the cross-sectional study, we were only able to determine sarcopenia and related factors. Cohort studies are needed to clarify the causal relationship between sarcopenia and its associated factors.

## 5. Conclusions

The prevalence of sarcopenia was high among community-dwelling older persons in Hunan Province. Old age and nutritional disorders were more likely to have sarcopenia. No falls in the previous year, high BMI, and a high level of physical activity were associated with a lower incidence of sarcopenia. Lifestyle is an important controllable factor for sarcopenia. We should explore intervention programmes that involve nutrition, exercise, and sleep to minimise the risk of sarcopenia.

## Figures and Tables

**Table 1:** Characteristics of older adults with or without sarcopenia.

Variables	Sarcopenia, 282 (27.1%)	Non-Sarcopenia, 758 (72.9%)	*p*-Value
Age (years)	73.62 ± 8.47	69.18 ± 6.56	<0.05
Female (No. and %)	187 (69.3%)	515 (67.9%)	>0.05
Height (m)	1.51 ± 0.07	1.58 ± 0.08	<0.05
Weight (kg)	48.51 ± 6.52	61.74 ± 8.70	<0.05
farmer (No. and %)	141 (53.3%)	354 (46.7%)	>0.05
**D** **egree**			<0.05
Below primary school (No. and %)	82 (29.1%)	175 (23.1%)	
Primary school (No. and %)	82 (29.1%)	226 (29.8%)	
Junior high (No. and %)	68 (24.1%)	209 (27.6%)	
Senior high (No. and %)	36 (12.8%)	122 (16.1%)	
University and above (No. and %)	14 (4.9%)	26 (3.4%)	
Married (No. and %)	195 (69.1%)	616 (81.3%)	<0.05
Falling in the past year (No. and %)	98 (12.9%)	59 (20.9%)	<0.05
Smoking (No. and %)	168 (22.2%)	71 (25.2%)	<0.05
Drinking (No. and %)	134 (17.7%)	50 (17.7%)	<0.05
BMI (kg/m^2^)	24.95 ± 5.67	21.22 ± 2.69	<0.05
**PASE**			<0.05
Low level of physical activity (No. and %)	104 (13.7%)	63 (22.3%)	
Middle physical activity level (No. and %)	494 (65.2%)	178 (63.1%)	
High level of physical activity (No. and %)	160 (21.1%)	41 (14.6%)	
**MNA-SF**			<0.01
Normal nutritional status (No. and %)	614 (51.8%)	140 (51.8%)	
Risk of malnutrition (No. and %)	146 (45.4%)	128 (45.4%)	
Malnutrition (No. and %)	6 (2.8%)	8 (2.8%)	
**SRSS**			<0.05
Normal sleep condition (No. and %)	407 (53.7%)	139 (49.3%)	
Mild sleep disorder (No. and %)	224 (29.6%)	91 (32.3%)	
Moderate sleep disorder (No. and %)	118 (15.6%)	47 (16.7%)	
Severe sleep disorders (No. and %)	9 (1.2%)	5 (1.8%)	

**Table 2:** Comparison of older adult-related indicators among different ages (*n* = 1040).

Variables	60–69 (*n* = 518)		70–79 (*n* = 387)		>80 (*n* = 135)		*p*-Value
	Sarcopenia	Non-Sarcopenia	Sarcopenia	Non-Sarcopenia	Sarcopenia	Non-Sarcopenia	
Prevalence (No. and %)	101 (19.5%)	417 (80.5%)	112 (28.9%)	275 (71.5%)	69 (51.1%)	66 (48.9%)	<0.01
SMI (kg/m^2^)	5.74 ± 0.66	6.75 ± 0.87	5.52 ± 0.80	6.86 ± 1.38	5.35 ± 0.78	6.77 ± 1.02	<0.01
Grip strength (kg)	21.42 ± 8.65	23.57 ± 8.00	16.31 ± 6.37	21.86 ± 8.52	13.02 ± 6.19	17.48 ± 8.99	<0.01
6 meters walking speed (m/s)	0.99 ± 0.31	1.01 ± 0.29	1.18 ± 0.41	1.17 ± 0.62	1.72 ± 0.86	1.65 ± 1.01	<0.01
CRT (s)	9.54 ± 3.34	9.71 ± 3.12	11.77 ± 4.52	11.23 ± 3.80	15.75 ± 5.96	14.45 ± 5.55	<0.01
BMI (kg/m^2^)	21.07 ± 2.37	24.50 ± 3.06	21.43 ± 2.93	21.64 ± 8.46	20.94 ± 2.99	24.80 ± 2.85	<0.01
Per cent body fat (%)	28.99 ± 7.83	31.99 ± 7.53	30.94 ± 7.94	32.77 ± 8.79	30.76 ± 8.90	33.16 ± 7.44	<0.01

**Table 3:** Comparison of elderly-related indicators among different genders (*n* = 1040).

Variables	Males (*n* = 337)		Females (*n* = 697)		*p*-Value
	Sarcopenia (*n* = 95)	Non-Sarcopenia (*n* = 243)	Sarcopenia (*n* = 187)	Non-Sarcopenia (*n* = 515)	
Prevalence (No. and %)	26.2%	73.8%	25.2%	74.8	>0.05
SMI (kg/m^2^)	6.38 ± 0.45	7.72 ± 1.26	5.14 ± 4.50	6.35±0.64	<0.01
Grip strength (kg)	23.54 ± 8.23	29.50 ± 8.35	14.18 ± 5.58	19.08 ± 6.12	<0.01
6 meters walking speed (m/s)	1.19 ± 0.64	1.12 ± 0.55	1.27 ± 0.57	1.13 ± 0.55	<0.01
CRT (s)	11.51 ± 5.46	10.41 ± 3.46	12.16 ± 4.15	10.81 ± 4.07	<0.01
BMI (kg/m^2^)	21.24 ± 2.86	25.30 ± 8.79	21.15 ± 2.71	24.77 ± 3.26	<0.01
Per cent body fat (%)	25.37 ± 7.77	26.87 ± 7.40	32.64 ± 7.24	34.97 ± 6.90	<0.01

**Table 4:** Multivariate regression analysis of influencing factors of sarcopenia in the elderly.

	β	SE	Walds Value	*p* Value	OR Value	95% CI
Constant	2.531	0.728	12.087	0.001	12.568	
Age	0.908	0.184	24.459	0.000	2.480	(1.730, 3.553)
Fall history	0.634	0.226	7.841	0.005	0.616	(1.885, 1.209)
**BMI**						
Underweight			94.860	0.000		
Normal weight	−1.479	0.375	15.594	0.000	0.228	(0.109, 0.475)
Overweight	−3.517	0.431	66.540	0.000	0.030	(0.013, 0.069)
**MNA-SF**						
Normal nutritional status			15.341	0.000		
The risk of malnutrition	0.735	0.189	15.149	0.000	2.085	(1.440, 3.019)
Malnutrition	0.192	0.706	0.074	0.785	1.212	(0.304, 4.834)
**PASE**						
Low physical activity			8.584	0.014		
Moderate physical activity	−0.522	0.231	5.118	0.024	0.593	(0.377, 0.933)
High physical activity	−0.874	0.303	8.328	0.004	0.417	(0.230, 0.755)

## Data Availability

The datasets used and analysed during the current study are available from the corresponding author upon reasonable request.
